# LncRNA GAS5 restrains ISO-induced cardiac fibrosis by modulating mir-217 regulation of SIRT1

**DOI:** 10.1038/s41598-024-58239-9

**Published:** 2024-04-01

**Authors:** Yan-hong Zhang, Ting-ting Sun, Zhen-hua Liu, Xu Li, Xiao-Fang Fan, Li-ping Han

**Affiliations:** 1 Institute of Hypoxia Medicine, School of Basic Medical Sciences, Wenzhou Medical University, Chashan Higher Education Park, Wenzhou, Zhejiang China; 2 Department of Pathology Changhai Hospital, Second Military Medical University, Shanghai, China; 3 Department of Physiology, School of Basic Medical Sciences, Wenzhou Medical University, Wenzhou, China

**Keywords:** GAS5, SIRT1, Mir-217, Myocardial fibrosis, Pyroptosis, Cardiovascular biology, Circulation, Cardiovascular biology

## Abstract

Considering the effect of SIRT1 on improving myocardial fibrosis and GAS5 inhibiting occurrence and development of myocardial fibrosis at the cellular level, the aim of the present study was to investigate whether LncRNA GAS5 could attenuate cardiac fibrosis through regulating mir-217/SIRT1, and whether the NLRP3 inflammasome activation was involved in this process. Isoprenaline (ISO) was given subcutaneously to the male C57BL/6 mice to induce myocardial fibrosis and the AAV9 vectors were randomly injected into the left ventricle of each mouse to overexpress GAS5. Primary myocardial fibroblasts (MCFs) derived from neonatal C57BL/6 mice and TGF-β1 were used to induce fibrosis. And the GAS5 overexpressed MCFs were treated with mir-217 mimics and mir-217 inhibitor respectively. Then the assays of expression levels of NLRP3, Caspase-1, IL-1β and SIRT1 were conducted. The findings indicated that the overexpression of GAS5 reduced the expression levels of collagen, NLRP3, Capase-1, IL-1β and SIRT1 in ISO treated mice and TGF-β1 treated MCFs. However, this effect was significantly weakened after mir-217 overexpression, but was further enhanced after knockdown of mir-217. mir-217 down-regulates the expression of SIRT1, leading to increased activation of the NLRP3 inflammasome and subsequent pyroptosis. LncRNA GAS5 alleviates cardiac fibrosis induced via regulating mir-217/SIRT1 pathway.

## Introduction

Heart failure (HF) is a major cause of mortality, hospitalizations, and reduced quality of life and a major burden for the healthcare system^1^. Despite great clinical advances and strategies during the past decades, heart failure continues to pose a major health concern worldwide. Cardiac remodeling, contributing to the pathophysiology of heart failure, is a chronic maladaptive process characterized by vascular dysfunction, myocardial hypertrophy, apoptosis, necrosis, ventricular dilatation, and myocardial fibrosis^2,3^. Cardiac fibrosis is characterized by increased collagen deposition and/or its altered composition in the myocardium^4,5^. It is manifested by the replacement of normal myocardium by non-functional fibrotic tissues^6^. The administration of renin–angiotensin–aldosterone system blockers and other drugs can only delay the development of myocardial fibrosis^7^. At present, there are no other effective means to reverse or prevent myocardial fibrosis.

Sirtuin 1 (SIRT1), also known as nicotine adenine dinucleotide-dependent deacetylase, has shown a strong protective role in cardiovascular diseases. SIRT1 may attenuate isoproterenol-induced cardiac fibrosis by regulating EndMT via the TGF-β/Smad2/3 pathway^8^. Drugs or interventions targeted at SIRT1 may become an important therapeutic scheme for preventing cardiac fibrosis^9^. A few studies have shown that a variety of miRNAs are involved in cardiovascular diseases via targeting SIRT1^10,11^.

MicroRNAs (miRNAs) are a class of highly conserved non-coding single-stranded RNAs (20–26 nucleotides in length) that regulate gene expression in eukaryotes. Bioinformatics analysis showed that SIRT1 has multiple microRNAs (mir-217, mir-140, mir-138, mir-204) binding sites. mir-217 is involved in a variety of cardiovascular diseases, i.e. aggravating hypoxia-induced myocardial cell injury via targeting SIRT1^12,13^.

Long non-coding RNAs are transcripts exceeding 200 nucleotides in length without functional protein-coding potential. LncRNAs are found to regulate gene expression at the post-transcriptional level in eukaryotic cells^14^. In the last decades, increasing evidence suggests that long non-coding RNAs (lncRNAs) serve as a critical regulator of cardiac physiological and pathological processes, regarded as a new target of treatment for heart failure^15^. Studies have shown that LncRNA growth arrest-special transcript 5 (GAS5) also plays an important role in cardiovascular diseases such as myocardial fibrosis^16^, while the mechanism remains unclear. However, it is clear that GAS5 can participate in the progression of cancer by targeting microRNA^17–19^, and mir-217 is also one of the target genes of GAS5^20^, which provides a theoretical basis for GAS5 to the regulation of myocardial fibrosis through mir-217/sirt1 pathway.

Pyroptosis is an inflammatory programmed cell death activated in response to microbial infection, cellular damage, or metabolic imbalances^21^. Oligomerization of NLRP3 inflammasome mediates the activation of caspase-1 and the release of pro-inflammatory cytokines IL-1β and IL-18^22^. Previous evidence suggests that NLRP3 inflammasome plays a vital role in the pathogenesis of cardiovascular diseases such as atherosclerosis, atrial fibrillation, and myocardial infarction^23–25^. A recent study also found that GAS5 is involved in regulating the pathophysiological process of diabetic cardiomyopathy caused by NLRP3 inflammasome activation^26^.

Therefore, the aim of the present study was to investigate whether LncRNA GAS5 could attenuate cardiac fibrosis through regulating mir-217/SIRT1, and whether the NLRP3 inflammasome activation was involved in this process. The regulatory effect of mir-217 on myocardial fibrosis in vitro was evaluated in Mouse Cardiac Fibroblasts (MCFs), and the relevant molecular mechanisms were determined. This study provides significant experimental evidence suggesting LncRNA GAS5 to be a potential therapeutic target for cardiac fibrosis and other fibrotic diseases.

## Results

### Overexpression of GAS5 improved cardiac function and alleviated cardiac fibrosis induced by isoproterenol (ISO) in C57BL/6 mice

**Figure 1:**
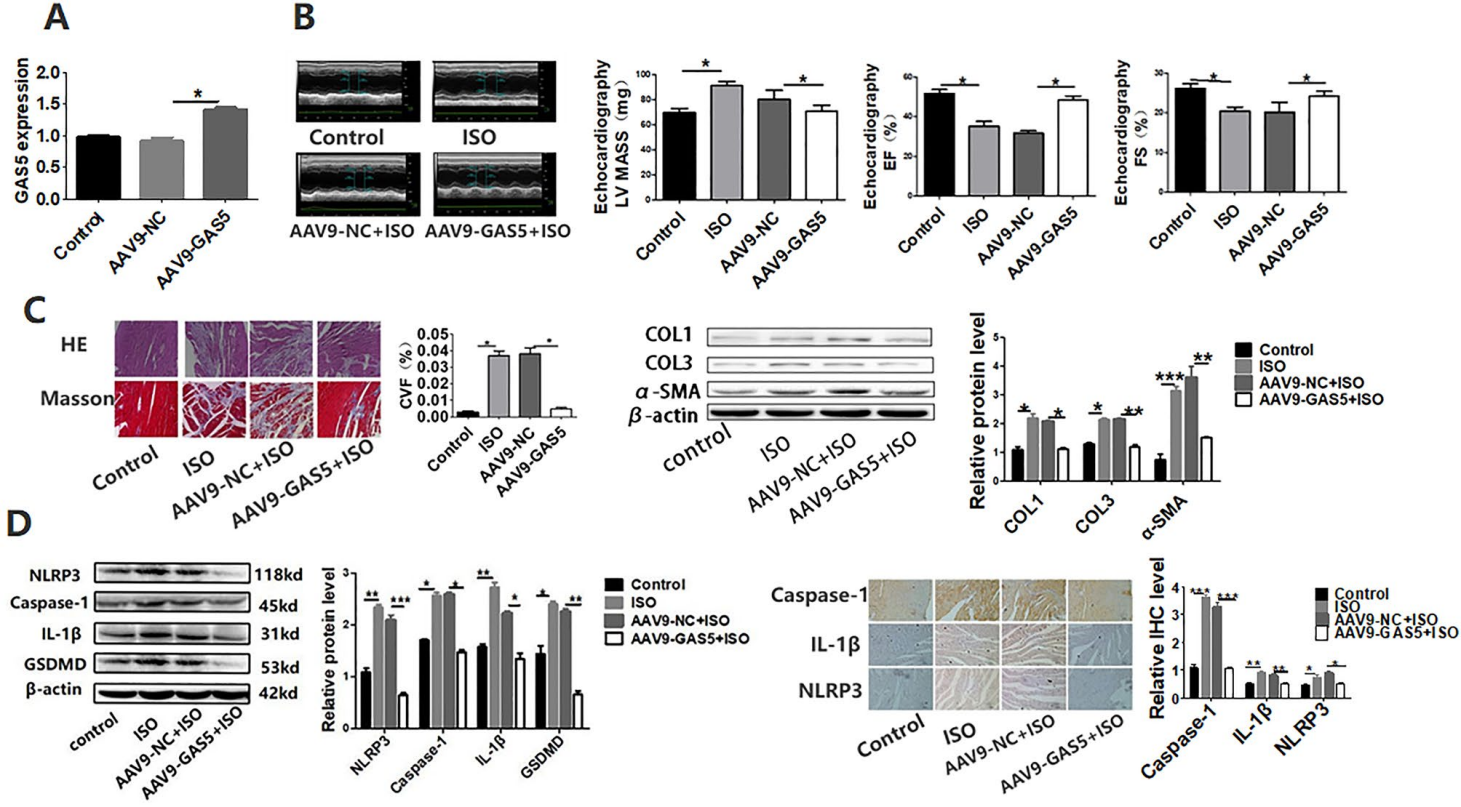
Overexpression of GAS5 improved cardiac function and alleviated cardiac fibrosis induced by isoproterenol (ISO) in C57BL/6 mice via inhibiting NLRP3 inflammasome activation-mediated pyroptosis. (**A**) C57BL/6 mice were transfected with ADV-GAS5 or ADV-NC. Normal mice served as control The expression of GAS5 in heart tissues was detected by RT-qPCR. (**B**) Overexpression of GAS5 improved cardiac ejection and attenuated cardiac hypertrophy induced by isoproterenol. Representative echocardiographic photos were from M-mode. Echocardiographic parameters included ejection fraction (EF), fractional shortening (FS) and left ventricular mass (LV mass). (**C**) Overexpression of GAS5 alleviated cardiac fibrosis induced by isoproterenol. The results were shown by HE and Masson’s trichrome staining of the left ventricular myocardium; Protein expression of collagen I, collagen III and α-SMA. (**D**) Overexpression of GAS5 inhibits NLRP3 inflammasome activation-mediated pyroptosis in vivo. WB and immunohistochemistry were performed to explore the expression of active forms of NLRP3, caspase-1, IL-1β and GSDMD in heart tissues. *p < 0.05, **p < 0.01, ***p < 0.001. Data are represented as mean ± SEM, *n* = 10.

To identify the function of GAS5 in cardiac fibrosis, we induced the cardiac fibrosis in GAS5-overexpressed mice by subcutaneous injecting isoproterenol (ISO) and investigated the cardiac function with echocardiography. Figure 1A showed that GAS5 was overexpressed in the mice’s hearts by AAV9-GAS5 transfection. As shown in Fig. 1B, the left ventricular (LV) mass is remarkably increased in ISO group with respect to the control group. Overexpression of GAS5 augmented LV ejection fraction (EF), and LV fractional shortening (FS). To measure the degree of cardiac fibrosis, histological analysis was performed using hematoxylin and eosin (HE) and Masson’s trichrome staining. The AAV9-NC + ISO group showed greater myofibril disarray and collagen deposition than did those in the AAV9-GAS5 + ISO group (Fig. 1C). Quantitative analysis of mRNA expression and western blot assay confirmed these results (Fig. 1C). These findings suggest that GAS5 overexpression alleviates cardiac fibrosis in vivo.

### Overexpression GAS5 inhibited NLRP3 inflammasome activation-mediated pyroptosis in vivo

To investigate whether GAS5 inhibits pyroptosis in cardiac fibrosis, WB and RT-qPCR were performed to explore the expression of NLRP3, caspase-1, IL-1β and GSDMD in heart tissues of mice. The mice hearts from ISO Group exhibited a pronounced increase in the expression of NLRP3, caspase-1, IL 1β and GSDMD as compared with the control group. The expression of these proteins was significantly inhibited in the AAV9-GAS5 + ISO group (Fig. 1D). Immunohistochemical results showed the same changes (Fig. 1D). These data imply that overexpression of GAS5 inhibits NLRP3 inflammasome activation-mediated pyroptosis in vivo.

### Overexpression of GAS5 suppressed the expression of mir-217 and promoted the expression of SIRT1 in vivo and vitro; GAS5 alleviated myocardial fibrosis and suppressed pyroptosis in vitro

**Figure 2:**
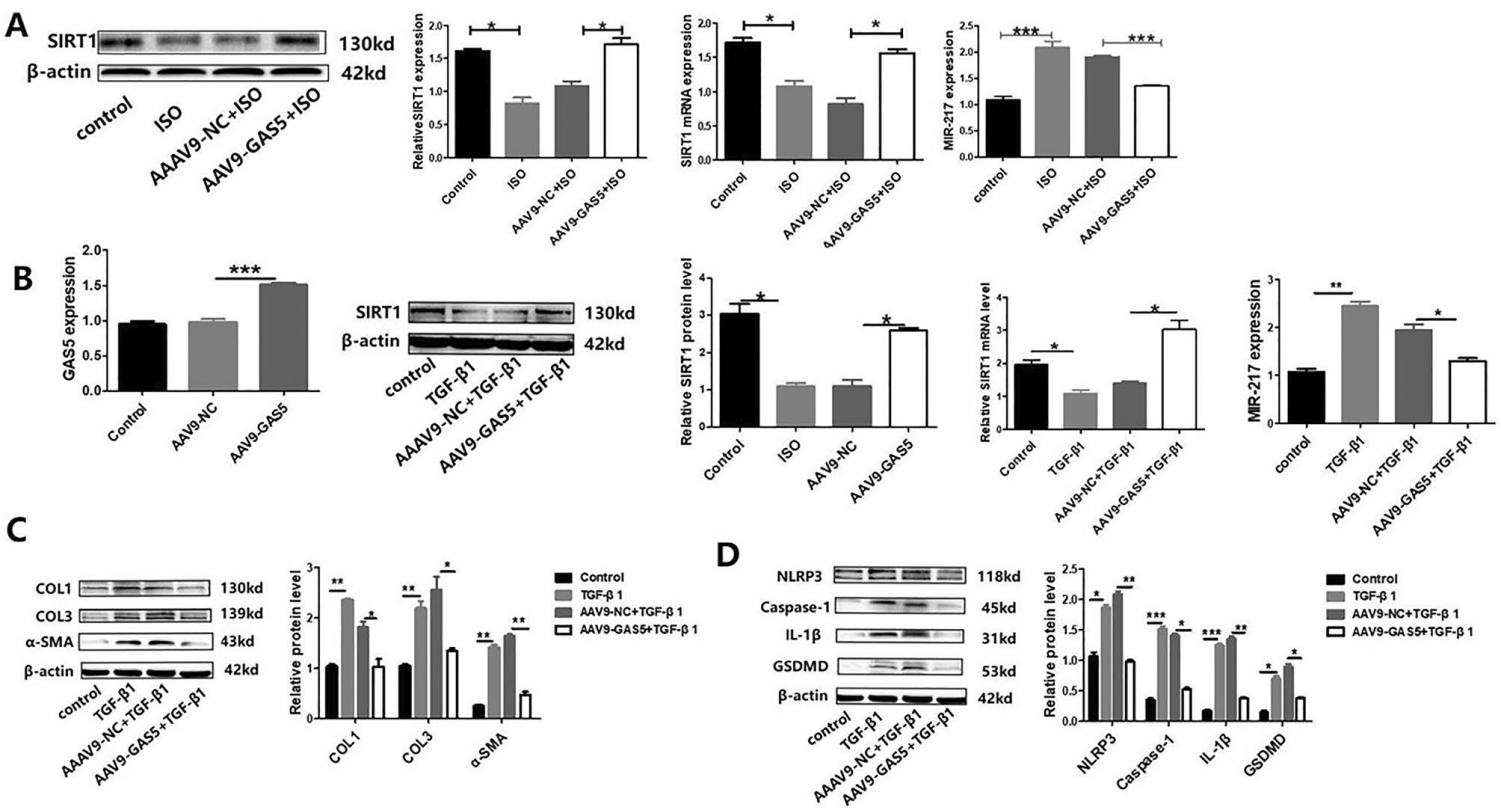
Overexpression of GAS5 suppressed the expression of mir-217 and promoted the expression of SIRT1 both in vivo and in vitro. (**A**) To prove that the overexpression of GAS5 suppressed the expression of mir-217 and promoted the expression of SIRT1 in vivo, WB and RT-qPCR were performed to explore the expression of SIRT1, and PCR was performed to explore the expression of mir-217. (**B**) To prove that the overexpression of GAS5 suppressed the expression of mir-217 and promoted the expression of SIRT1 in vitro, MCFs were transfected with AAV9-GAS5 or AAV9-NC. Then the expression of GAS5 and mir-217 in MCFs was detected by RT-qPCR; the expression of SIRT1 was detected by RT-qPCR and WB. (**C**) Protein expression of collagen I, collagen III and α-SMA showed that the overexpression of GAS5 alleviated fibrosis of MCFs induced by TGF-β1. (**D**) Protein expression of NLRP3, Caspase-1, IL-1β and GSDMD showed that the overexpression of GAS5 suppressed pyroptosis in the TGF-β1-treated MCFs. *p < 0.05, **p < 0.01, ***p < 0.001. Data are represented as mean ± SEM, *n* = 6.

Bioinformatics data reveal that GAS5 and mir-217 have highly binding sequences. So we overexpressed GAS5 in mice, then we found that the overexpression of GAS5 suppressed mir-217 and promoted SIRT1 expression in vivo (Fig. 2A). To further examine the effect of GAS5 on mir-217 and SIRT1 in vitro, AAAV9-GAS5 were used to overexpress GAS5 in MCFs and fibrosis was induced by TGF-β1. As shown in Fig. 2B, overexpression of GAS5 was achieved successfully. WB assay and RT-qPCR analysis revealed that GAS5 regulated the expression of mir-217 and SIRT1 in the same way as in vivo (Fig. 2B). These data imply that overexpression of GAS5 inhibited the expression of mir-217 and promoted the expression of SIRT1. The expression of collagen I, collagen III and α-SMA are remarkably increased in TGF-β1-treated MCFs (Fig. 2C). But the expression of these proteins was significantly inhibited by GAS5 overexpression. Meanwhile, quantitative analysis of mRNA expression and western blot assay showed the expression of NLRP3, Caspase-1, IL-1β and GSDMD changed in the same way (Fig. 2C). These findings suggest that overexpression of GAS5 alleviates fibrosis and pyroptosis in vitro.

### mir-217 aggravated fibrosis via NLRP3 inflammasome activation-mediated pyroptosis in vitro

**Figure 3:**
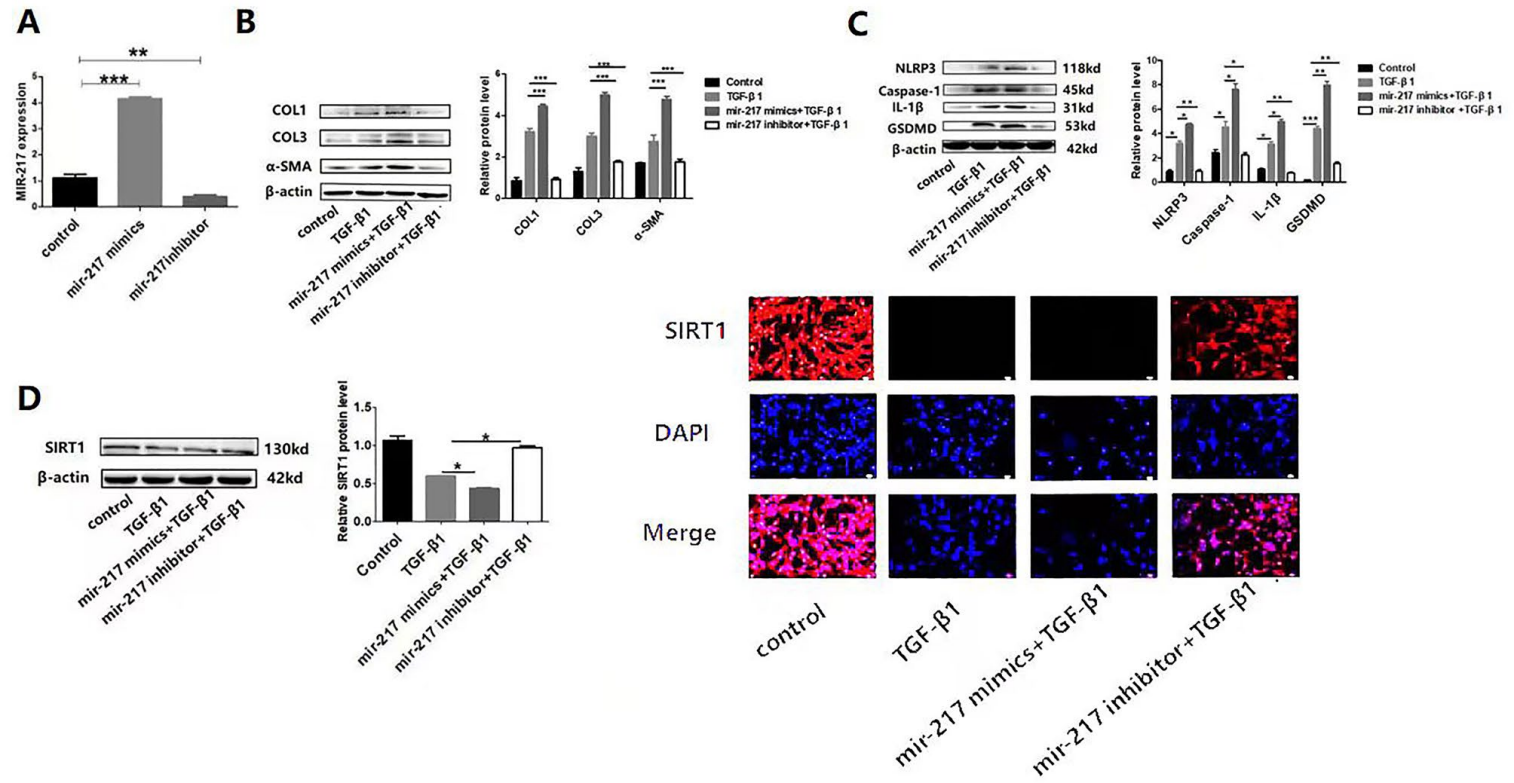
mir-217 aggravated fibrosis via down-regulating the expression of SIRT1 but stimulating NLRP3 inflammasome activation-mediated pyroptosis in vitro. (**A**) MCFs were transfected with mir-217 mimics and inhibitor. Then the expression of mir-217 in MCFs was detected by RT-qPCR. (**B**) Protein expression of collagen I, collagen III and α-SMA in MCFs was detected by WB. (**C**) Protein expression of NLRP3, Caspase-1, IL-1β and GSDMD in MCFs was detected by WB. (**D**) Protein and mRNA expression of SIRT1 in MCFs was detected, and immunofluorescence staining of SIRT1(red) was performed in MCFs at × 200 magnification. *p < 0.05, **p < 0.01.**P < 0.01, ***P < 0.01. Data are represented as mean ± SEM, n = 6.

To investigate whether mir-217 could exacerbate pyroptosis in cardiac fibrosis, fibrosis was induced by treatment with TGF-β1 and overexpression or underexpression of mir-217 were achieved by treatment with mir-217 mimics or inhibitor (Fig. 3A). In mir-217 mimics + TGF-β1 group, the expression of collagen I, collagen III and α-SMA rose remarkably compared with TGF-β1 group. The expression of these proteins was notably inhibited in mir-217 inhibitor + TGF-β1 group (Fig. 3B). Quantitative analysis of mRNA expression and western blot assay revealed that the expression of NLRP3, Caspase-1, IL-1β and GSDMD changed in the same way (Fig. 3C). These findings suggest that mir-217 aggravates myocardial fibrosis and NLRP3 inflammasome activation-mediated pyroptosis in vitro.

### mir-217 down-regulated the expression of SIRT1.

To further demonstrate that mir-217 plays a key role in myocardial fibrosis and pyroptosis via SIRT1, expression of SIRT1 was assayed. The results showed that the expression of SIRT1 was remarkably increased in mir-217 mimics + TGF-β1 group with respect to TGF-β1-treated MCFs. But the expression of SIRT1 was notably inhibited in mir-217 mimics + TGF-β1 group (Fig. 3D).

### LncRNA GAS5 alleviated cardiac fibrosis induced by NLRP3 inflammasome activation-mediated pyroptosis via regulating mir-217/SIRT1 pathway

**Figure 4:**
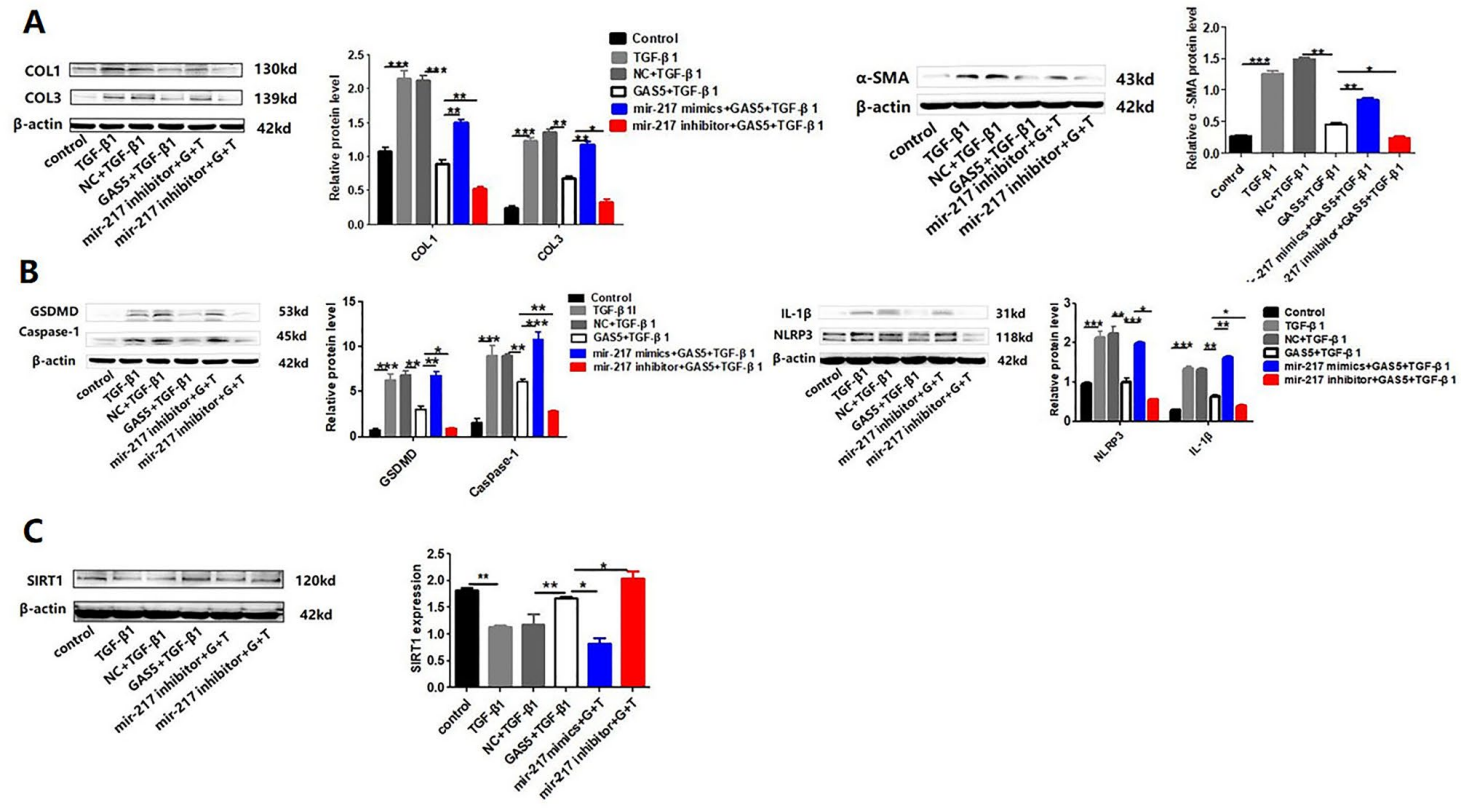
LncRNA GAS5 alleviateed cardiac fibrosis induced by NLRP3 inflammasome activation-mediated pyroptosis via regulating mir-217/SIRT1 pathway. The MCFs were co-transfected with GAS5 overexpression plasmid and mir-217 mimics or inhibitor, respectively, then were treated with TGF-β1. Then the protein expression of collagen I, collagen III and α-SMA (**A**); the protein expression of NLRP3, Caspase-1, IL-1β and GSDMD (**B**); and the protein and mRNA expression of SIRT1 (**C**) in MCFs were detected. *p < 0.05, **p < 0.01.**P < 0.01, ***P < 0.01. Data are represented as mean ± SEM, n = 6.

According these evidences shown above, it can be confirmed that GAS5 overexpression could inhibit the expression of mir-217, then promote the expression of SIRT1 in vivo and vitro. And mir-217 could down-regulate the expression of SIRT1 then aggravate myocardial fibrosis induced by NLRP3 inflammasome activation-mediated pyroptosis in vitro. To further demonstrate the mechanism by which GAS5 regulates myocardial fibrosis, MCFs were co-transfected with GAS5 overexpression plasmid and mir-217 mimics or inhibitor, respectively, then were treated with TGF-β1. As shown in Fig. 4, the overexpression of GAS5 reduced the expression levels of collagen, NLRP3, Capase-1, IL-1β and SIRT1 in TGF-β1 group. However, these effects were significantly weakened by mir-217 overexpression while these effects were further enhanced by knockdown of mir-217.

## Discussion

Evidence suggests that microRNA plays an important intermediary role in the mechanism of GAS5 regulating the occurrence and development of cardiovascular diseases^27^. And experiments performed on primary myocardial fibroblasts showed that overexpression of GAS5 could inhibit fibrosis in vitro^28^. To study the effects of GAS5 on myocardial fibrosis in vivo, we overexpressed GAS5 in mice and found that GAS5 did significantly elevate the pumping function of mice hearts treated with Isoprenaline (ISO). The overexpression of GAS5 also alleviated cardiac fibrosis induced by ISO in mice.

Pyroptosis is characterized by the formation of the nucleotide-binding domain (NOD)-like receptor protein 3 (NLRP3) inflammasome complex that is composed of NLRP3, apoptosis-associated speck-like protein (ASC), and pro-caspase-1^29^. Inflammasome is a group of cytosolic multiprotein complexes including NLRP1, NLRP2, NLRP3, AIM2, and NLRC4. Among them, the NLRP3 inflammasome has been widely reported in studies of tumor and neurological diseases. The NLRP3 inflammasome can be activated by a variety of stresses with multiple activation pathways including microRNAs, long-chain non-coding RNA (lncRNAs)^30–32^. And it has been shown that GAS5 is involved in renal tubular cell pyroptosis^33^, ovarian cancer^34^ and diabetic cardiomyopathy (DCM)^35^ induced by NLRP3 inflammasome activation. Previous studies have shown that pyroptosis activation is associated with Cardiac Dysfunction^36^. In the current study, we tested the expression of NLRP3, Caspase-1, IL-1β and GSDMD which are pyroptosis markers. The results showed that the expression of these markers was significantly increased in the mice hearts treated with ISO, while the overexpression of GAS5 inhibited this increase of these markers expression induced by ISO.

The evidences mentioned above imply that GAS5 may inhibit myocardial fibrosis via regulating NLRP3 inflammasome activation, but the exact mechanism requires further investigations. The regulatory mechanisms of GAS5 involved in the pathophysiological process of cardiovascular diseases are very complex, and one of them is to regulate various microRNAs and their downstream target genes^37–39^. Increasing studies have reported that lncRNAs play crucial functions in numerous biological processes by interacting with the 3′-untranslated region (UTR) of target mRNAs to silence their expression^40^. It is well-known that SIRT1 probably provides a new therapeutic regimen for preventing heart failure and plays an important role in the occurrence and development of myocardial fibrosis. Study showed that therapeutic strategies to restore appropriate SIRT1 signaling could mitigate ongoing fibrogenesis^41^. Previous studies have shown that microRNA targets SIRT to regulate pyroptosis of diseases^42,43^ and mir-217 could negatively regulate expression of SIRT1^44^. Our study in mice found that the overexpression of GAS5 inhibits the expression of mir-217 and promotes the expression of SIRT1. Therefore we hypothesized that GAS5 could regulate the downstream target gene SIRT1 via mir-217 and subsequently inhibit myocardial fibrosis.

Transforming Growth factor β (TGF-β) is a central trigger of myocardial fibroblast activation that regulates a wide variety of biological processes such as extracellular matrix (ECM) production, cell growth, differentiation, cell homeostasis and migration^45–47^. And it can directly enhance collagen expression to induce fibrotic diseases^48^. In order to prove our hypothesis, we treated the MCFs with TGF-β1 to induce fibrosis after co-transfection of GAS5 overexpression plasmid and mir-217 mimics or mir-217 inhibitor. The results showed that mir-217 negatively regulated SIRT1 and aggravated the myocardial fibrosis induced by TGF-β1 via NLRP3 inflammasome activation. And the overexpression of GAS5 inhibited the myocardial fibrosis and the NLRP3 inflammasome activation. The mir-217 mimics offset this effect of GAS5 on myocardial fibrosis, pyroptosis and SIRT1, and the mir-217 inhibitor enhanced all these effects of GAS5. It can be concluded that GAS5 inhibits myocardial fibrosis induced by NLRP3 inflammasome activation through the mir-217/SIRT1 pathway. Our study is the first to demonstrate that mir-217 played a vital role in developing myocardial fibrosis through regulating pyroptosis.

Our study and some recent articles have demonstrated that there is a functional connection between GAS5 and mir-217^20,49^. We constructed the wild type and variant type vectors of GAS5 based on a highly bound sequence between GAS5 and mir-217, and performed dual luciferase gene assay. The experimental results were negative (RiboBio, Guangzhou). However, target prediction between GAS5 and mir-217 revealed the existence of three highly bound sequences between GAS5 and mir-217, and GAS5 is a long-chain non-coding RNA. So, the negative result may be caused by the diversity of transcripts. Therefore, it cannot be entirely concluded that there is no structural interaction between GAS5 and mir-217. New vectors for dual luciferase gene assay are required to be constructed to verify the structural interaction between GAS5 and mir-217 in future studies.

## Materials and methods

### Reagents

Isoprenaline (ISO) was purchased from Sigma-Aldrich (St. Louis, MO, USA) and dissolved in phosphate-buffered saline (PBS). TGF-β1 (100-21) was obtained from Peprotech (Rocky Hill, NJ, USA). mir-217 mimics and mir-217 inhibitor were purchased from Hanbio Biotechnology Co., Ltd. (Shanghai, China). Antibody against α-SMA was purchased from Abcam (Cambridge, MA, USA). The following antibodies were purchased from Proteintech (Chicago, IL, USA): SIRT1, collagen I, collagen III, GSDMD, Caspase-1, IL-1β, NLRP3 and β-actin. AAV9-GAS5 (recombinant adeno-associated virus expressing growth arrest specific transcript-5) and AAV9-NC were constructed by Genechem (Shanghai, China).

### Animals and treatment

The study is reported in accordance with ARRIVE guidelines. Adult male C57BL/6 mice, aged 8 ~ 10 weeks, were purchased from the Wenzhou Medical University Laboratory Animal Centre (Wenzhou, China). The Animal Certificate NO is SYXK (浙) 2010-0044. All animal-procedures were performed in accordance with the Guide for the Care and Use of Laboratory Animals published by the US National Institutes of Health (NIH Publication No. 85-23, revised 1996). The animals were housed individually in cages under hygienic conditions in a 12/12-h light/dark cycle at 22 ± 3 °C and 45 ± 10% humidity for seven days before the experiments. The study was evaluated and approved by the Wenzhou Medical University Animal Care and Use Ethics Committee (permission number wydw2013-0054). The animals were allowed free access to a standard commercial diet and tap water. Before and after the experiment, all of the mice were treated humanely. Then the mice were randomly divided into four groups (10 per group) for treatment: Control, ISO, AAV9-NC + GAS5, and AAV9-GAS5 + ISO. In the ISO model mice, ISO was injected subcutaneously at 15 mg/kg/day for three days and then at 10 mg/kg/day for 11 days^8,50^. In AAV9-NC group and AAV9-GAS5 group, AAV9 vectors at a dose of 1 × 10^11^ viral genome particles were randomly injected into the left ventricle of each mouse. Less than 20 μL AAV9 vectors were injected into the heart at 5 sites. The RT-qPCR results verified the successful mouse cardiac GAS5 overexpression after two weeks, followed by ISO treatment, which is the same as ISO group. In control group, the mice were injected subcutaneously with the same dose of sterile saline.

### Echocardiographic analysis of cardiac function

The mice were lightly anesthetized with 1.5% isoflurane in a medical air/oxygen mixture during examinations and placed supine on a heated platform with electrocardiography leads. Heart images were acquired using a Vevo1100 system (VisualSonics System, Toronto, Ontario, Canada). Measurements were made three consecutive times, over at least 10 cardiac cycles each time. The following parameters were recorded: ejection fraction (EF), fractional shortening (FS), and left ventricular mass (LV mass).

### Histological quantification of cardiac fibrosis

After the 14-day treatment described above, all mice were sacrificed and the hearts were quickly excised, arrested in diastole with 1.34 M KCl, weighed, placed in 4% paraformaldehyde, and embedded in paraffin. The hearts were cut transversely close to the apex. Sections of the heart (4 ~ 5 μm) were mounted onto slides and stained with hematoxylin and eosin (H&E, Solarbio, Beijing, China) for histopathological analysis. The tissue sections were also subjected to Masson’s trichrome staining (Solarbio, Beijing, China) and visualized by light microscopy (ECLIPSE 80i, Nikon Corporation) to determine collagen deposition. The photographs were analyzed using digital analysis software (ImageJ) in a blinded manner.

### Histological analysis of NLRP3 inflammasome activation-mediated pyroptosis

For immunohistochemical analysis, cardiac sections were deparaffinized in xylene and rehydrated in a graded ethanol series. Endogenous peroxidase activity was quenched in 3% methanol-H_2_O_2_ for 10 min. Antigen retrieval was performed by heating the sections in 10 mM citrate buffer (pH 6.0) in a microwave oven. Non-specific binding was blocked in 1% bovine serum albumin. Next, the sections were incubated with primary antibodies against Caspase-1, IL-1β, and NLRP3 at 4 °C overnight and washed three with PBS for 5 min per wash. The sections were then incubated with second antibodies for 30 min before nuclear staining. The results were analyzed by fluorescence microscopy (ECLIPSE Ti-S; Nikon Corporation).

### Cell culture and treatments

Primary myocardial fibroblasts (MCFs) were isolated from the hearts of 3-day-old neonatal male C57BL/6 mice as previously described^51^, which were obtained from the laboratory animal center of Wenzhou Medical University. MCFs were cultured in 1640 medium supplemented with 10% fetal bovine serum, 100 U/mL penicillin, and 100 U/mL streptomycin (Sigma, Milan, Italy) at 37 °C in a humidified atmosphere containing 5% CO_2_. After overnight serum starvation, MCFs were randomly grouped into: Control, TGF-β1, TGF-β1 + AAV9-NC and TGF-β1 + AAV9-GAS5.

#### Adeno-associated virus infection

MCFs in the logarithmic growth phase were selected and detached with trypsin. Then the cells were cultured on 96-well plates in complete culture medium (1640 + 10%FBS) at 37 °C in a humidified atmosphere containing 5% CO_2_ for 24 h. The adeno-associated virus was added and infected cells in 1/2 volume of the same medium (1640 + 10%FBS). The number of viruses was calculated according to the optimal MOI (10^6^). 1/2 volume of fresh culture fluid was added 4 h later to continue infect at 37 °C for 48 h. The treated cells were divided into two groups: Group A was AAV9-NC and Group B was AAV9-GAS5. According to the optimum MOI, Adeno-associated virus were added to complete medium for culture before cells were infected with virus. Cell passage and plating were initiated when cell density developed to 80%. Then the cells were divided into four groups: Control, TGF-β1, AAV9-NC + TGF-β1, AAV9-GAS5 + TGF-β1. The cells were collected with Trizol and RT-qPCR was performed to verify the viral infection.

#### Co-transfection cell experiment

DEPC water was added into the GAS5 overexpressed plasmid or NC so that its final concentration is 100 nM, then use 1640 medium to prepare 20 mM for reserve. 3 ~ 12 × 10^15^ MCFs were seeded into 150 μL sterile medium 24 h before transfection. When the cell fusion rate reached 30% ~ 50%, the transfection was initiated. 5 μL GAS5 plasmid was diluted with 250 μL 1640 medium to make 50 nM transfection concentration. Transfection reagent Lipofectanine TM2000 was diluted and mixed with the diluents of GAS5 overexpression or NC plasmid. The mixed reagent was added to the 6-well plate containing the cell solution for culture. The transfection effect was verified by RT-qPCR and then successfully transfected MCFs were subcultured. Cell passage and plating were initiated when cell density was 80%. 3 ~ 12 × 10^15^ GAS5 overexpressed MCFs were seeded into 150 μL sterile medium 24 h before the transfection. When the cell fusion rate reached 30% ~ 50%, transfection was initiated. 100 nM mir-217 mimics and inhibitor were diluted with 1640 medium to 20 mM for reserve. Transfection reagent was diluted and mixed with the diluents of mir-217 mimics and mir-217 inhibitor. The mixed reagent was added to the 6-well plate containing the cell solution for culture. Then the cells were divided into six groups: Control, TGF-β1, NC + TGF-β1, GAS5 + TGF-β1, mir-217 mimics + GAS5 + TGF-β1, mir-217 inhibitor + GAS5 + TGF-β1.

### Western blot

Heart tissue and MCFs were lysed in RIPA protein extraction buffer (Applygen, Beijing, China) and centrifuged at 4 °C at 12,000 rpm for 15 min. The supernatant was collected and protein quantification was carried out by a bicinchoninic acid protein assay (Beyotime, Shanghai, China). The extracted protein was separated with sodium dodecyl sulfate–polyacrylamide gel electrophoresis (SDS-PAGE) and transferred onto polyvinylidene fluoride membranes. After being blocked with 5% skimmed milk in TBST at room temperature for 1 h, the membranes were incubated with primary antibodies at 4 °C overnight, anti-collagen I (1:1000, Proteintech), anti-collagen III (1:1000, Proteintech), anti-SIRT1 (1:1000, Proteintech), anti-GSDMD (1:1000, Proteintech), anti-caspase-1 (1:1000, Proteintech), anti-β-actin (1:5000, Proteintech), anti-IL-1β (1:1000, HuaAn), anti-NLRP3 (1:1000, HuaAn), anti-α-SMA (1:1000, Abcam). Following washing three times with TBST, the membranes were incubated with a secondary antibody for 1 h at room temperature, then washed three more times with TBST. Protein bands were visualized in ECL and the targeted proteins were scanned with the Molecular Imager ChemiDoc XRS Imaging System (Bio-Rad, Hercules, CA, USA) (Supplementary figure).

### Quantitative real-time polymerase chain reaction (RT-qPCR)

The mRNA levels of collagen I, collagen III, α-SMA, NLRP3, IL-1β, and GSDMD were analyzed by RT-qPCR. RNA was extracted from heart tissues and MCFs using Trizol reagent following the manufacturer’s instructions. The RNA purity was evaluated based on the OD260/OD280 ratios with micronuclei acid instrument. The RNA was reverse-transcribed into cDNA using the Takara Kit, Gene CopoeiaTM Kit. Diluted primers, samples, and RNA Free ddH_2_O were dissolved on ice and fully mixed and centrifuged. The PCR was performed via TIANGEN Kit and Gene CopoeiaTM Kit. The primers used in the study are shown in the Table 1.

**Table 1:** Gene-specific primers used in quantitative real-time polymerase chain reaction (RT-qPCR).

Species	Genes	Sequences
Mouse	GAS5	Forward: 5′-GGATAACAGAGCGAGCGCAAT-3′ Reverse: 5′-CCAGCCAAATGAACAAGCATG-3′
Mouse	SIRT1	Forward: 5′-GCTGACGACTTCGACGACG-3′ Reverse: 5′-TCGGTCAACAGGAGGTTGTCT-3′
Mouse	α-SMA	Forward: 5′-TCCCTGGAGAAGAGCTACGAACT-3′ Reverse: 5′-AAGCGTTCGTTTCCAATGGT-3′
Mouse	Collagen I	Forward: 5′-GCTCCTCTTAGGGGCCACT-3′ Reverse: 5′-CCACGTCTCACCATTGGGG-3′
Mouse	Collagen III	Forward: 5′-CTGTAACATGGAAACTGGGGAAA-3′ Reverse: 5′-CCATAGCTGAACTGAAAACCACC-3′
Mouse	GAPDH	Forward: 5′-AGGTCGGTGTGAACGGATTTG-3′ Reverse: 5′-TGTAGACCATGTAGTTGAGGTCA-3′
Mouse	NLRP3	Forward: 5′-GTGGAGATCCTAGGTTTCTCTG-3′ Reverse: 5′-CAGGATCTCATTCTCTTGGATC-3′
Mouse	IL-1β	Forward: 5′-CCCTGCAGCTGGAGAGTGTGG-3′ Reverse: 5′-TGTGCTCTGCTTGAGAGGTGCT-3′
Mouse	Caspase 1	Forward: 5′-ACACGTCTTGCCCTCATTATCT-3′ Reverse: 5′-ATAACCTTGGGCTTGTCTTTCA-3′
Mouse	GSDMD	Forward: 5′-TGCACCACCAACTGCTTAG-3′ Reverse: 5′-GGATGCAGGGATGATGTTC-3′
Mouse	mir-217	Forward: 5′-TACTGCATCAGGAACTGACTGGA-3′
Mouse	U6	Forward: 5′-GATGACACGCAAATTCGTG-3′

### Statistical analysis

The results are expressed as mean ± SEM and were analyzed by one-way analysis of variance (ANOVA) and Tukey's post-hoc test (*p < 0.05 and **p < 0.01) with GraphPad Prism 5.0 software.

### Ethics approval

All animal experimental protocols in this study were approved by the Animal Care and Use Committee of Laboratory Animal Centre of Wenzhou Medical University.

### Consent to participate

Yes, my co-authors and I would like to participate.

### Supplementary Information

Supplementary Figures.
