# Supplementary figures and images for: LncRNA GAS5 restrains ISO-induced cardiac fibrosis by modulating mir-217 regulation of SIRT1
Source: Sci Rep. 2024 Apr 1;14:7652. doi: 10.1038/s41598-024-58239-9 (PMC10985102; doi:10.1038/s41598-024-58239-9)

**
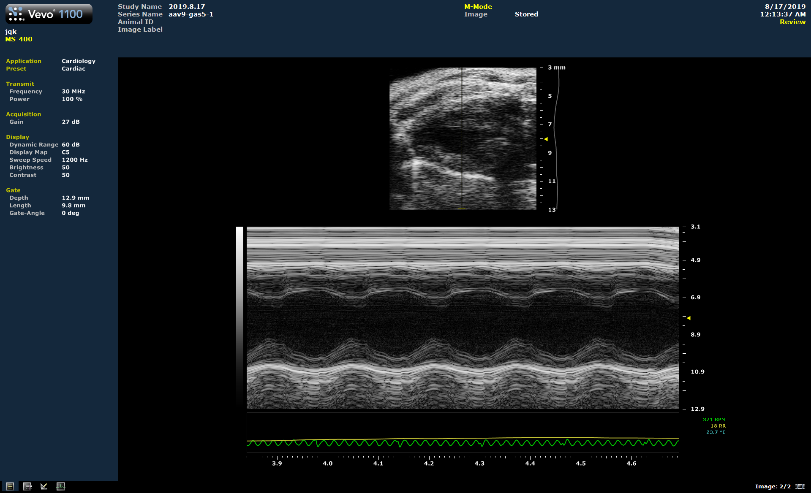

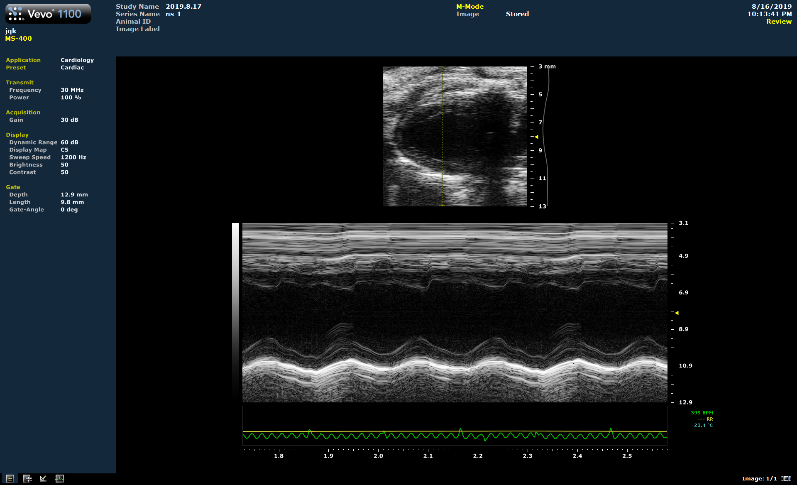

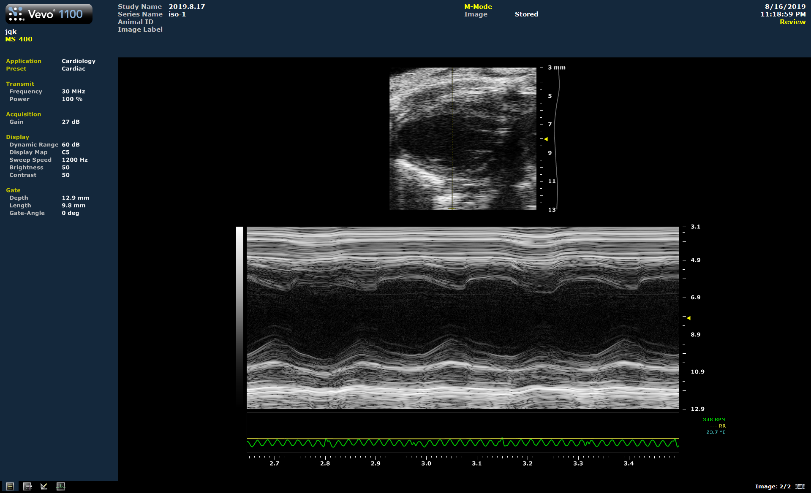

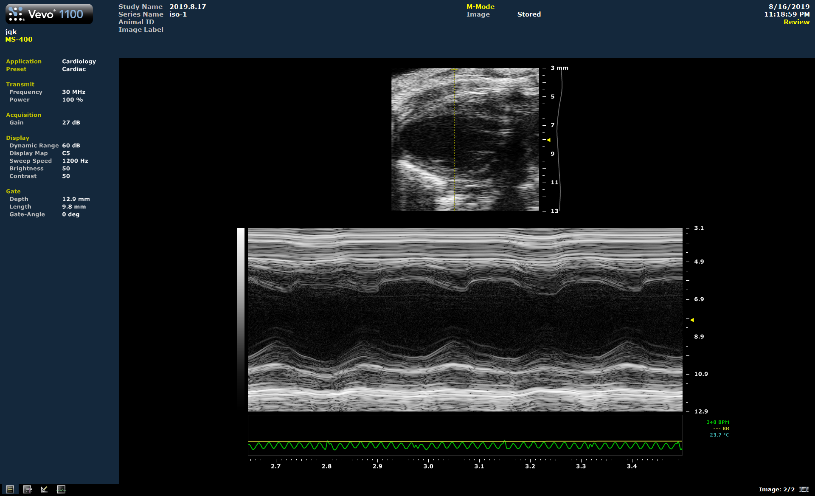
1B**

**AAV9-GAS5**

**AAV9-NC**

**ISO**

**Control**

**
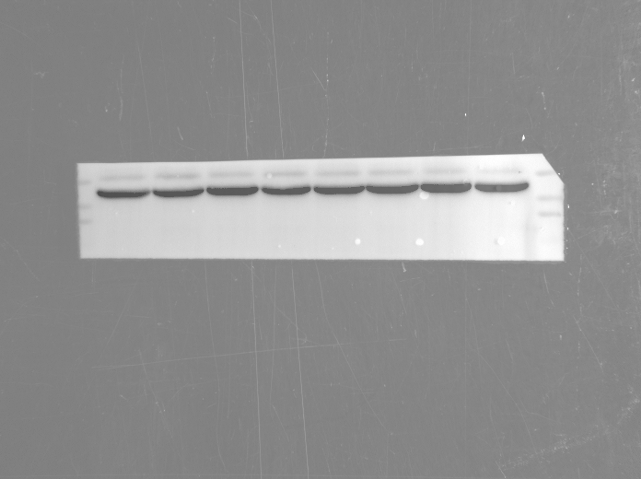

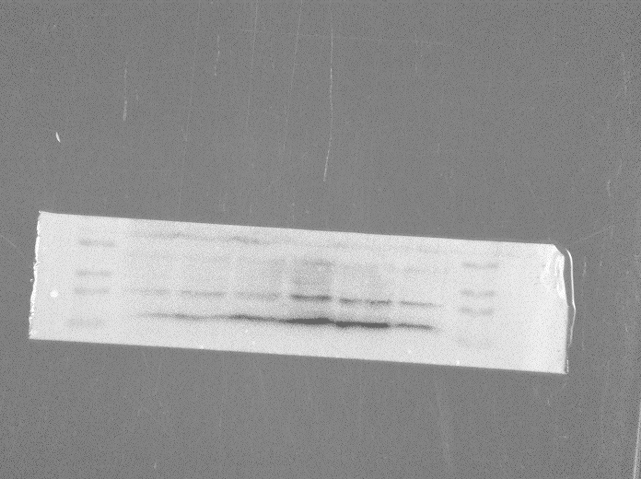

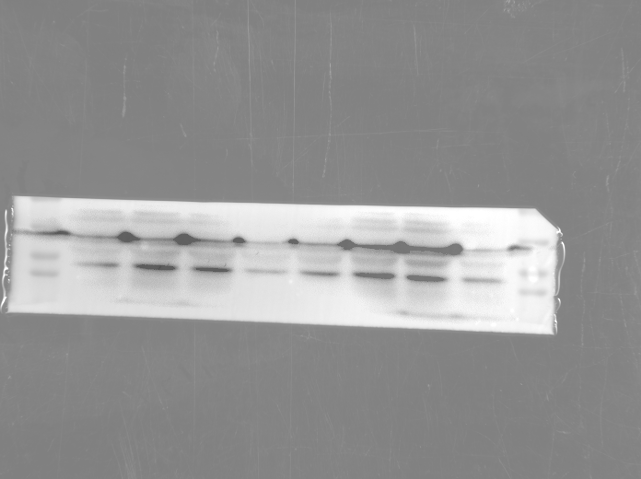

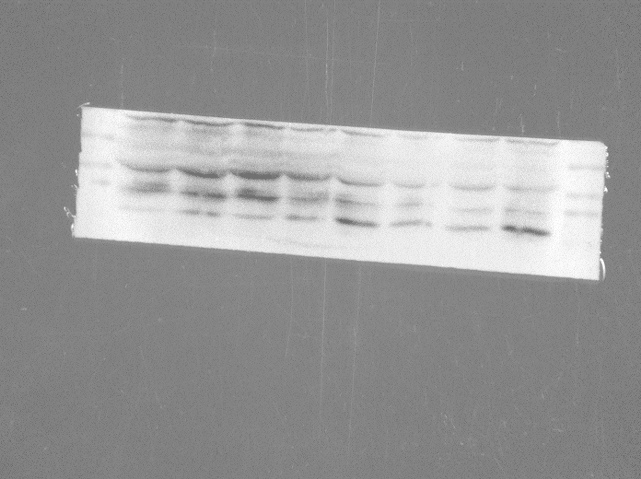

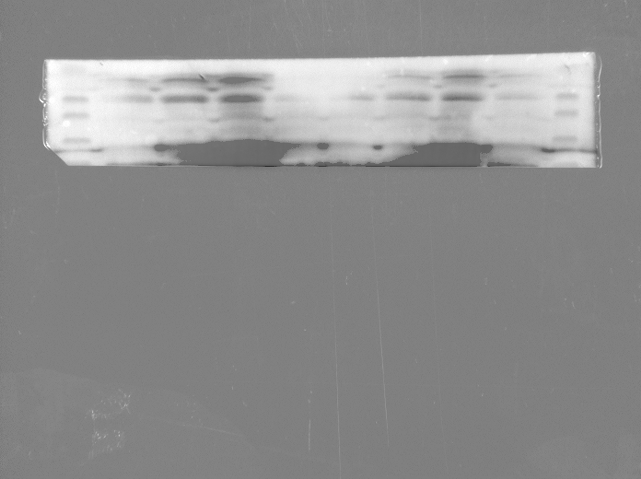
1D**

**β-actin**

**GSDMD**

**IL-1β**

**NIRP3**

**Caspase-1**

Supplement: Supplementary file 1 — Supplementary Figures. [file 41598_2024_58239_MOESM1_ESM.docx]
